# Identifying pediatric neurosurgery training priorities in Paraguay: a novel quadrant analysis

**DOI:** 10.1007/s00381-025-07064-0

**Published:** 2025-12-03

**Authors:** Victor M. Lu, Mauricio Guerrero-Ocampo, Ivan García, Luis M. Peña, Italo Flecha, Kevin Arce, Sima Vazquez, Caleigh S. Roach, Diego Servián

**Affiliations:** 1 Department of Neurological Surgery, Hospital General Pediátrico “Niños de Acosta Ñu”, San Lorenzo, Paraguay; 2 Department of Neurological Surgery, University of Miami Miller School of Medicine, Jackson Memorial Hospital, 1095 NW 14th Terrace, Miami, Florida 33136 USA; 3 Department of Neurological Surgery, Hospital de Clinicás-Universidad Nacional de Asunción, San Lorenzo, Paraguay

**Keywords:** Pediatric, Neurosurgery, Paraguay, Middle-income, LMIC, Global neurosurgery, Priority

## Abstract

**Background:**

The landscape of pediatric neurosurgical training in Paraguay is largely unknown, partly due to the absence of pediatric fellowship training in the country. Correspondingly, the aim of this study was to identify priorities for future training initiatives using a novel quadrant analysis based on current trainee perceptions.

**Methods:**

Paraguayan neurosurgical trainees were surveyed regarding their familiarity with multiple pediatric neurosurgical diseases (*n* = 21), procedures (*n* = 24), and technologies (*n* = 10). Familiarity was scored on a discrete numerical scale. The mean and standard deviation of pooled values were then plotted in a quadrant analysis as importance and urgency, respectively, to identify priority groups for future training endeavors.

**Results:**

A total of 23 Paraguayan neurosurgical trainees, including 14 of the total 15 (93%) active Paraguayan neurosurgical residents, completed this survey. The majority of respondents believed there was insufficient time in residency to adequately train in pediatric neurosurgery (*n* = 19, 83%). The most common barriers reported were cost (*n* = 14, 61%) and availability (*n* = 9, 39%). Our quadrant analysis showed that in terms of pediatric neurosurgical disease understanding, the highest priority was spasticity, and the lower priorities included hydrocephalus and infection. In terms of pediatric cranial and spine procedures, the highest priorities were found to be non-peritoneal ventricular shunts, endoscopic lavage, vertebral body tethering (WBT), and selective dorsal rhizotomy (SDR). Finally, in terms of technology, the highest priorities were intraoperative electrocorticography (ECOG), laser ablation, and focused ultrasound (FUS). All respondents were open to more dedicated pediatric exposure as part of their residency training.

**Conclusions:**

Our novel quadrant analysis identified multiple diseases, procedures, and technologies that are priorities for future pediatric neurosurgery training initiatives in Paraguay. Further, this approach can likely be used similarly in other middle- and low-income countries to continue to raise the bar of global pediatric neurosurgery training.

## Introduction

Paraguay is a landlocked South American country with a population of 6.8 million inhabitants. Per the World Bank, it is considered an Upper Middle Income Country (UMIC); however, its gross domestic product (GDP) ranks amongst the lowest in South America, with its Human Development Index (HDI) ranking 105th in the world [1–3]. Reviews on the state of pediatric neurosurgical training in countries with these socioeconomic metrics are likely to have limitations [4, 5]. To date, the current landscape and priority areas in pediatric neurosurgery training within Paraguay have not been investigated.

Neurosurgical training in Paraguay is centered around the capital, Asunción, and is spread across three primary tertiary sites that treat both adult and pediatric patients. There is no dedicated pediatric rotation at any of these sites, and trainees are exposed to pediatrics on an ad hoc basis. As such, exposure to the spectrum of pediatric neurosurgery for any trainee is highly dependent on hospital volume, reach, and attending expertise [6]. *Hospital General Pediátrico "Niños de Acosta Ñu"* is the only dedicated pediatric hospital in all of Paraguay, located within the Asunción locale, At this hospital, although there is a dedicated neurosurgery service led by dedicated specialists, there is no formal resident or trainee involvement.

The current organization of neurosurgery training in Paraguay suggests that there are likely training gaps and areas to improve pediatric neurosurgical training in Paraguay. Correspondingly, the aim of this study was to survey current trainees in Paraguay to identify priorities for future training initiatives using a novel quadrant analysis based on current trainee perceptions.

## Methods

### Survey cohort

A cross-sectional anonymous survey was created consisting of multiple choice and free-form short answer questions in Spanish. The survey was conducted during the endorsed ‘*Pre-Congress Neuroendoscopy Course*’ prior to the national Paraguayan Neurosurgical Society Meeting in June 2025. Trainees for this study were defined as both active residents in neurosurgical residency and recent graduates within their first year of graduation. All residents from the country’s three training programs were given permission to attend this course by the national neurosurgical society, allowing for high resident representation in this survey. Recent graduates were also invited to attend the course.

### Familiarity scoring

Trainee familiarity was assessed for multiple pediatric neurosurgical diseases (*n* = 21), procedures (*n* = 24), and technologies (*n* = 10). A 3-point Likert scale was used to assess familiarity– not familiar, familiar, very familiar. The decision to use these trichotomous outcomes was to avoid intrinsic uncertainty in further discriminative levels of familiarity. These values were assigned numerical values of 0, 1, and 2 to allow for quantitative analysis of mean and standard deviation (SD). The mean value represented how familiar each individual, on average, was. The SD value represented then how consistent the trainees were in their level of familiarity.

### Quadrant analysis

A novel quadrant analysis was employed to analyze the results of the survey. This approach was based on the known Eisenhower Matrix, a 2 × 2 matrix that compares the perceived importance versus urgency of tasks to triage priority [7]. Each quadrant represents then a level of priority. Those tasks of high importance/high urgency are considered the highest priority (Priority Group 1), followed by high importance/low urgency (Priority Group 2), then low importance/high urgency (Priority Group 3), then low importance/low urgency (Priority Group 4) [8].

This matrix and its variations have been used previously in the setting of clinical training to better triage and prioritize training components, including specialties such as general surgery [9], pediatrics [10], radiology [11], and emergency medicine [12]. Our study represents the first attempt to use this approach in the setting of neurosurgery. For our purposes, we appropriated importance and urgency with the statistical central tendency (mean and standard deviation (SD) values) of the familiarity scores. We defined higher importance as lower familiarity mean – implying a greater importance to teach and train the trainees about the parameters. We defined higher urgency as lower familiarity standard deviation – implying a more homogenous (lack of) understanding amongst trainees about the parameters that they were either familiar or not with. The use of quantitative mean and SD to help define the quadrant analysis has been previously reported in the broader behavioral science literature; however, it remains a novel concept in the setting of clinical medicine [13, 14]. All statistics were performed and visualized using Prism 10.0 (GraphPad, US).

## Results

### Cohort

A total of 23 respondents completed the survey (Table 1) – this was made up of 14 (61%) residents and 9 (39%) recent graduates from all three training sites in Paraguay. This included 14 of the total 15 (93%) active Paraguayan neurosurgical residents, There were 16 (70%) males and 7 (30%) females, and the most common age groups of respondents were 26–30 years and 31–35 years (both *n* = 11, 48%). A total of 15 (65%) respondents were interested in performing pediatric neurosurgery as part of their future practice.

**Table 1:** Characteristics of respondent cohort (*n* = 23) including demographics and summary of pediatric experience during training

Characteristic	Count (*n*, % total)
Demographics	
Gender	
Male	16 (70%)
Female	7 (30%)
Age group (yrs)	
20–25	1 (4%)
26–30	11 (48%)
31–35	11 (48%)
Training level	
Resident	14 (61%)
Recent graduate	9 (39%)
Interest in performing pediatric neurosurgery after training	
Yes	15 (65%)
No	8 (35%)
Pediatric experience	
Dedicated pediatric rotation	
Yes	0
No	23 (100%)
Number of pediatric cases participated in to date	
0–25	3 (13%)
26–50	4 (17%)
51–75	1 (4%)
75–100	1 (4%)
Above 100	14 (61%)
Belief sufficient time in residency for pediatric training	
Yes	4 (17%)
No	19 (83%)
Pediatric mentor accessible	
Yes	8 (35%)
No	15 (65%)

### Current pediatric training experience

At time of survey, 14 (61%) respondents had participated in more than 100 pediatric neurosurgery cases, which included all recent graduates (Table 1). No respondent reported dedicated pediatric training time in their 5-year residency – rather, all reported that they were exposed to pediatric neurosurgery throughout their whole residency at their primary training site. The majority of respondents declared that they believed this exposure be insufficient to learn pediatric neurosurgery (*n* = 19, 83%) and that they did not have access to a pediatric neurosurgery mentor (*n* = 15, 65%). The most common resources used by residents for pediatric neurosurgery were online materials (*n* = 23, 100%) followed by textbooks (*n* = 20, 87%), conferences (*n* = 15, 65%) and courses (*n* = 10, 43%). Multiple barriers to training opportunities for pediatric neurosurgery were reported. Cost (*n* = 14, 61%) was the biggest barrier reported, followed by availability (*n* = 9, 39%), case volume (*n* = 6, 26%), and awareness (*n* = 2, 9%).

### Familiarity with pediatric neurosurgery

All parameters were analyzed based on their assigned familiarity values and divided into their respective priority quadrant group (Table 2). The diseases with the highest and lowest familiarity scores in terms of understanding were congenital hydrocephalus (mean 1.87 ± 0.07) and spasticity (mean 0.57 ± 0.12), respectively. The cranial procedures with the highest and lowest familiarity scores were ventriculoperitoneal shunt (mean 1.91 ± 0.06) and neuroendoscopic lavage (NEL, mean 0.39 ± 0.10), respectively. The spine procedures with the highest and lowest familiarity scores were lumbar drain (mean 1.69 ± 0.11) and vertebral body tethering (VBT, mean 0.04 ± 0.02), respectively. The technologies with the highest and lowest familiarity scores were microscope (mean 1.91 ± 0.07) and laser ablation and focused ultrasound (mean 0.00 ± 0.00), respectively.

**Table 2:** Summary of priority groups based on familiarity of pediatric neurosurgical diseases, procedures, and technologies

	Priority Group 1	Priority Group 2	Priority Group 3	Priority Group 4
Diseases	• Spasticity	• AVM • Cavernoma • Chiari malformation • Epilepsy • Moyamoya disease • Scoliosis • Spine trauma • Stroke • Tethered cord	• Craniosynostosis • Spine tumor	• Abscess • Congenital hydrocephalus • Cranial trauma • Cranial tumor • Empyema • IVH • Meningitis • Myelomeningocele • Non-congenital hydrocephalus
Procedures (cranial)	• VA/VPl shunts • NEL	• Epilepsy craniotomy • ETV/CPC • Ommaya reservoir • Vascular craniotomy	• Craniosynostosis repair • ICP monitor • Tumor craniotomy	• EVD • Infection craniotomy • Trauma craniotomy • VP shunts
Procedures (spine)	• Vertebral body tethering	• Scoliosis instrumentation • Tethered cord release • SDR • Brachial plexus repair	• Myelomeningocele repair • Trauma instrumentation • Chiari malformation • Infection laminectomy • Tumor laminectomy	• Lumbar drain
Technologies	• ECOG • Focused ultrasound • Laser ablation	• Intraoperative ultrasound • Neuromonitoring • Neuronavigation • Robotic stereotaxy • Tubular system	• Endoscopy	• Microscope

*AVM* arteriovenous malformation, *IVH* intraventricular hemorrhage, *VA/VPl shunts* ventriculo-atrial/ventriculo-pleural shunts, *NEL* neuro-endoscopic lavage, *ETV/CPC* endoscopic third ventriculostomy/choroid plexus cauterization, *ICP monitor* intracranial pressure monitor, *EVD* external ventricular drain, *VP shunt* ventriculo-peritoneal shunt, *SDR* selective dorsal rhizotomy, *ECOG* electrocorticography

**Fig. 1:**
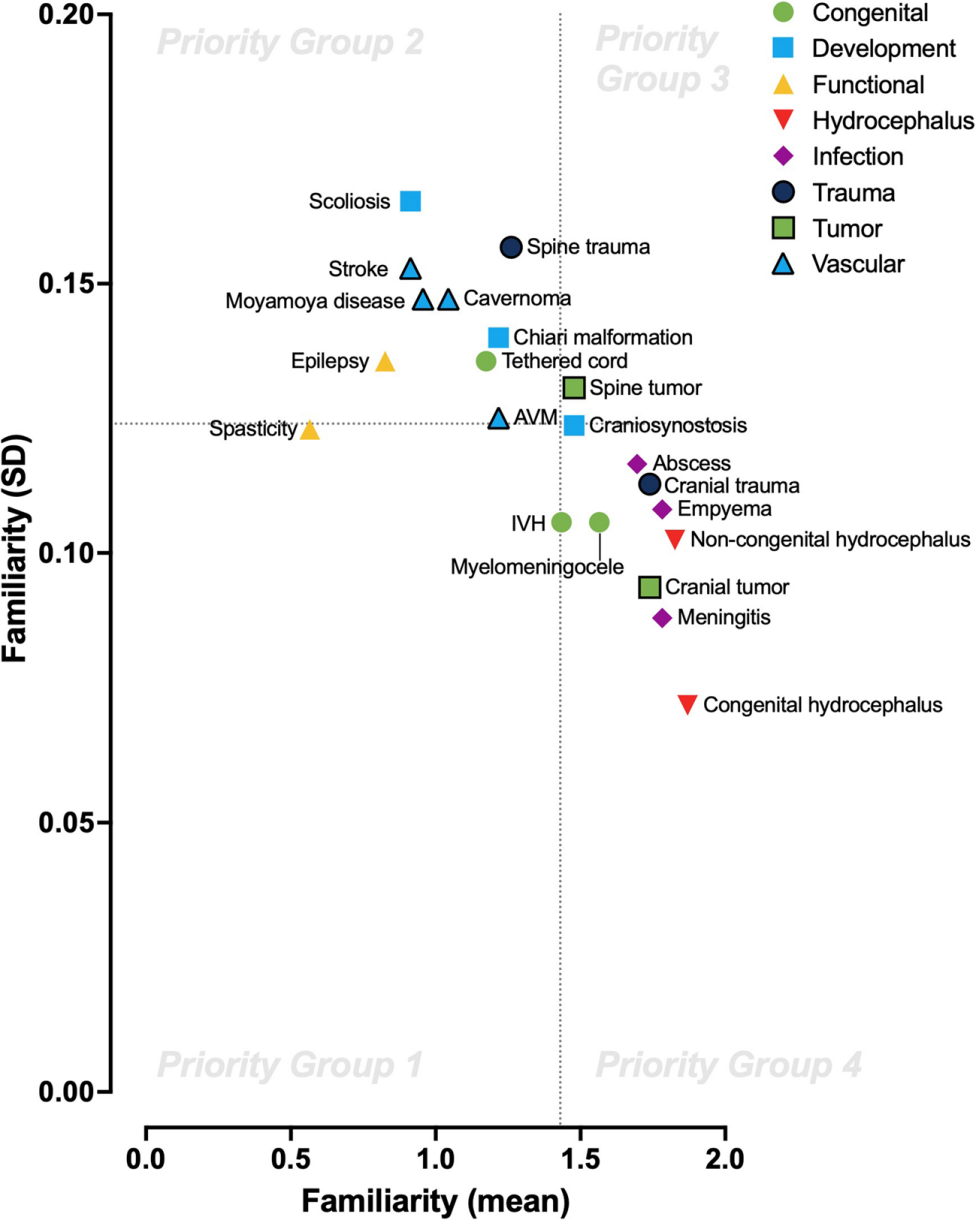
Familiarity plot of *n* = 21 pediatric neurosurgical diseases (mean versus SD). AVM, arteriovenous malformation; IVH, intraventricular hemorrhage

**Fig. 2:**
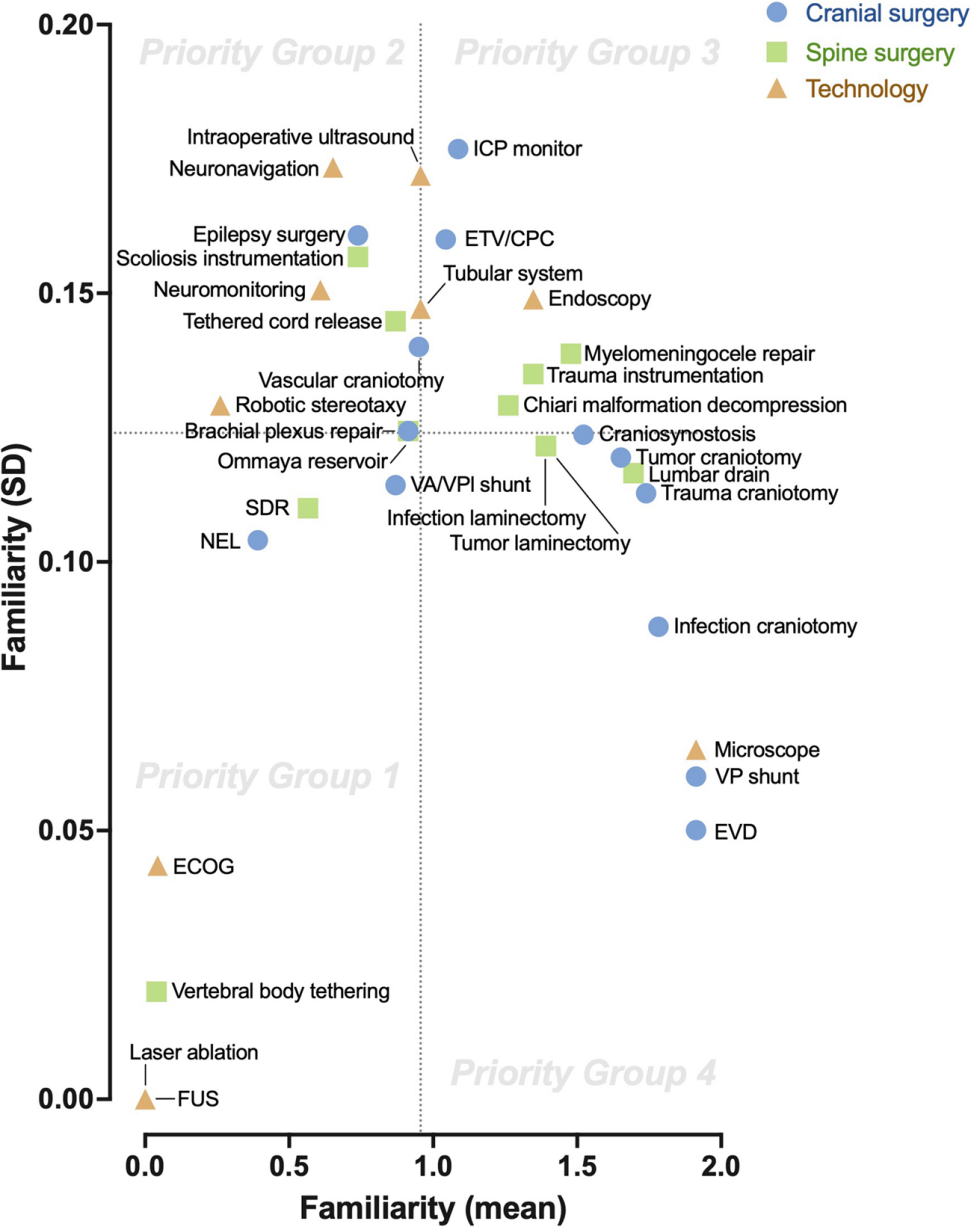
Familiarity plot of pediatric neurosurgical procedures (*n* = 24) and technologies (*n* = 10) (mean versus SD). VA/VPl shunts, ventriculo-atrial/ventriculo-pleural shunts; NEL, neuro-endoscopic lavage; ETV/CPC, endoscopic third ventriculostomy/choroid plexus cauterization; ICP monitor, intracranial pressure monitor; EVD, external ventricular drain; VP shunt, ventriculo-peritoneal shunt; SDR, selective dorsal rhizotomy; ECOG, electrocorticography

For disease understanding, median mean and SD familiarity values were 1.435 and 0.124, respectively (Fig. 1). As a result, Priority Group 1 consisted of spasticity. For surgical procedures and technologies, median mean and SD familiarity values were 0.956 and 0.124, respectively (Fig. 2). As a result, for cranial procedures, Priority Group 1 consisted of NEL and non-peritoneal ventricular shunts; for spinal procedures, Priority Group 1 consisted of VBT and selective dorsal rhizotomy (SDR); and for technologies, Priority Group 1 consisted of intraoperative electrocorticography, laser ablation, and focused ultrasound.

## Discussion

This study is the first survey of the pediatric training experience in Paraguay, and also additionally the first attempt to use a novel quadrant analysis method to identify elements in pediatric neurosurgery that should be prioritized in the training and exposure for interested trainees. For example, our data demonstrate the areas of functional, spine, and cranial endoscopy to be areas Paraguayan trainees would benefit from more exposure to in their pediatric training. Although anecdotal, these findings highlight how survey data and our modified 2 × 2 Eisenhower matrix can be harnessed to assist both local and international efforts to identify areas to improve the state of pediatric neurosurgical training in Paraguay.

Our survey findings parallel the reported lack of pediatric subspecialty training in neurosurgery in various other middle- and low-income countries. For example, in Ethiopia, there exists no local pediatric fellowship opportunity, and as such, advanced cranial and spine procedures, such as endoscopy, are largely uncommon in training or practice [15]. Another example in Africa is in Kenya, where the most common sources of teaching pediatric neurosurgery were online resources, as was in Paraguay [16]. Within South America, in Bolivia, which borders Paraguay, there is also a lack of vascular and functional surgeries being performed in pediatric centers, which directly impacts familiarity at the end of training [17].

Our quadrant analysis represents the first use of a novel modified Eisenhower matrix to identify training priorities in neurosurgery. The original matrix and its variations have been used previously in the setting of clinical training to better triage and prioritize its various components, including specialties such as general surgery [9], pediatrics [10], radiology [11], and emergency medicine [12]. What makes our findings unique is the comprehensive survey evaluation for multiple different parameters of pediatric neurosurgery training to be reported in the literature. We examined the familiarity of residents with the most common diseases one would encounter in a pediatric practice. It is not surprising that the most familiar diseases encountered are hydrocephalus, infection (meningitis), and neural tube defects, diseases more commonly associated with South American and African middle and low-income countries than elsewhere in the Western world [4, 16, 18, 19].

Inversely, our framework was also able to identify areas of least familiarity to these trainees –functional diseases such as spasticity, spine conditions such as scoliosis, and vascular diseases such as stroke. This may be in part rationalized by the fact that the diagnosis modalities and surgical management for these specific conditions are resource-intensive and costly. A global survey found that high-income countries had statistically significantly greater availability of pediatric epilepsy technology compared to other income groups, directly impacting training and familiarity [20]. For example, in African training programs, awareness and costs have limited the training of deep brain stimulation techniques for functional diseases [21]. In countries such as Paraguay where access to technology and treatment is limited, there is likely then a correlation to a more limited training experience [22].

We examined various surgical procedures to better understand what should be prioritized to improve clinical pediatric training in neurosurgery in Paraguay. Similar to disease familiarity, the majority of procedures of highest priority were functional (SDR, epilepsy), spine (scoliosis, vertebral body tethering, tethered cord) related, and endoscopic techniques (NEL, ETV/CPC). These areas mirror previous analyses of pediatric neurosurgery needs, which showed epilepsy surgery, spinal instrumentation, and endoscopy to be areas of the most need in both South American countries and LMICs, of which Paraguay is both [23]. Future studies are required to understand how much of this deficit in familiarity is a result of reduced disease prevalence versus a lack of infrastructure to support these procedures in a residency setting.

Given the majority of respondents were open to performing pediatric procedures in their future practice, our findings give direction to future initiatives to improve the standard of training in the future – whether that be targeted locally by specialists in Paraguay or targeted invitation of specialists outside of Paraguay to teach and share expertise on the areas that are less familiar. That is to say, local and foreign neurosurgeons may be able to run more focused in-person and electronic teaching sessions on the less familiar diseases, mission trips may choose to target procedures that are less familiar to residents, and charitable initiatives could target improving the availability of less familiar technologies.

A primary strength of this study is that it was able to capture effectively the entire active resident cohort within the country of Paraguay (14 of the 15 active residents responded). This is partly due to the fact that Paraguay only has three neurosurgery residency programs, all based around the capital Asunción. As such, the findings of this study are truly applicable to the entire Paraguayan training system.

There are limitations to this study. Firstly, there are intrinsic biases that may overestimate the familiarity of some conditions and procedures based on the fact it was an in-person survey [24]. Although the surveys were anonymous, the fact that the respondents were there in person may have prompted them to overestimate their familiarity due to fear of judgement. Secondly, the statistical grouping of priority in this study was anecdotal. It is difficult to predict whether or not the heterogeneity and parsing of our results would improve with more levels of familiarity than the three-tiered outcomes we employed. However, given the relatively small (although comprehensive) number of respondents available, it was thought that introducing further levels of familiarity would have only skewed the data. Future validation across other cohorts is envisaged to validate this approach.

## Conclusion

The landscape of pediatric neurosurgical training in Paraguay currently is not well understood. This is partly because there is limited pediatric exposure in the current training structure. Our quadrant analysis was able to identify specific pediatric neurosurgical diseases, procedures, and technologies as priorities in future training endeavors. This survey-based approach to identify training gaps has the potential to apply to other middle- and low-income countries, and future validation is required and encouraged.

## Data Availability

Available upon request.
